# Genome Sequencing Providing Molecular Evidence of Tetrapolar Mating System and Heterothallic Life Cycle for Edible and Medicinal Mushroom *Polyporus umbellatus* Fr.

**DOI:** 10.3390/jof11010015

**Published:** 2024-12-28

**Authors:** Shoujian Li, Youyan Liu, Liu Liu, Bing Li, Shunxing Guo

**Affiliations:** 1 The Institute of Medicinal Plant Development, Chinese Academy of Medical Sciences & Peking Union Medical College, Beijing 100193, China; 13756298450@163.com (S.L.); y1095192105@163.com (Y.L.); liuliu0026@foxmail.com (L.L.); zudengtianxia@126.com (B.L.); 2 State Key Laboratory of Bioactive Substance and Function of Natural Medicines, Chinese Academy of Medical Sciences and Peking Union Medical College, Beijing 100193, China

**Keywords:** sclerotia, fruiting body, nuclei migration, mating-type loci

## Abstract

*Polyporus umbellatus* is a species whose sclerotia have been extensively employed in traditional Chinese medicine, which has diuretic, antitumor, anticancer, and immune system enhancement properties. However, prolonged asexual reproduction has resulted in significant homogenization and degeneration of seed sclerotia. In contrast, sexual reproduction has emerged as an effective strategy to address these challenges, with a distinct mating system serving as the foundation for the implementation of sexual breeding. This study presents the first sequencing and assembly of the genome of *P. umbellatus*, thereby providing an opportunity to investigate the mating system at the genomic level. Based on the annotated mating-type loci within the genome, monokaryotic offspring exhibiting different mating-types were identified. Through the integration of traditional mating tests, the tetrapolar mating system of *P. umbellatus* was distinctly elucidated. The resequencing of monokaryotic strains with four different mating-types, along with comparative analyses of mating-type loci, revealed the *HD1* and *HD2* (*HD*, homeodomain) genes determined the mating *A* types, and the *PR4*, *PR5*, and *PR6* (*PR*, pheromone receptor) genes determined the mating *B* types. Meanwhile, this study offers a successful case study in the molecular investigation of mating systems. Additionally, the number of sterigma and basidiospores on each basidium was examined using scanning electron microscopy, while the nuclei of basidiospores and basidia at various developmental stages were analyzed through DAPI staining. This research clarifies the heterothallic life cycle of *P. umbellatus*. The findings of this study are expected to facilitate advancements in genetic research, breeding development, strain improvement, and the industry of *P. umbellatus*.

## 1. Introduction

*Polyporus umbellatus* (Pers.) Fr. is a member of the Polyporaceae family within the Basidiomycota phylum (https://www.indexfungorum.org/, accessed on 8 November 2024). This wood-decay fungus is widely distributed across the temperate regions of the Northern Hemisphere [1]. In China, the sclerotia of *P. umbellatus* are referred to as “Zhuling” and have been utilized in traditional medicine for over 2500 years [2]. They are also documented in various editions of the Chinese Pharmacopoeia [3]. The fruiting body of *P. umbellatus* consists of numerous caps that arise from a common stem [4,5]. In China, this mushroom is known as “Zhuling Mo”, which translates to “Zhuling mushroom”, and is recognized as a rare edible species. Recent advancements in pharmaceutical research have highlighted various bioactive properties of *P. umbellatus* sclerotia. In addition to its traditional diuretic effects [3], it has been reported to possess protective effects against renal fibrosis [6], anticancer properties [7], and antiviral activity [8].

The discovery of various medicinal properties has led to an increased demand for the sclerotia of *P. umbellatus*. Consequently, the excessive harvesting of this limited natural resource poses a threat to the survival of *P. umbellatus* [9]. Currently, this species has been included in the China Red Book since 2008 [10] and is also listed in the Red Data Book of Ukraine [11]. Additionally, it is recognized as endangered in Croatia, the Czech Republic, Hungary, Latvia, and Norway, while it is classified as threatened in Denmark, Finland, Great Britain, Sweden, and The Netherlands [12]. Fortunately, artificial cultivation of *P. umbellatus* was successfully initiated in China over 40 years ago [13], providing a new source of medicinal materials. Currently, the sclerotia of *P. umbellatus* are widely cultivated in the provinces of Shaanxi, Shanxi, Henan, Sichuan, and Yunnan in China [14]. It is estimated that the annual yield of *P. umbellatus* sclerotia in China is approximately 3000 tons.

Sexual reproduction enables organisms to generate novel combinations of alleles, thereby enhancing the potential fitness of their offspring [15]. However, the sclerotia of *P. umbellatus* can only be cultivated by utilizing the sclerotia as to produce new sclerotia through asexual reproduction. Prolonged asexual cultivation of sclerotia has resulted in significant homogenization and degeneration of the seed sclerotia. Unfortunately, the fruiting bodies of *P. umbellatus* are formed only sporadically, despite their potential to produce sexual offspring. The conditions for the artificial induction of fruiting bodies in *P. umbellatus* remain unclear. To date, only Pasailiuk (2020) has reported the artificial cultivation of *P. umbellatus* fruiting bodies, although no mature or normal fruiting bodies were observed [12]. Recently, successful crossbreeding has been achieved through the use of conidia of *P. umbellatus*, providing a new avenue for sexual breeding [16].

The distinct mating system serves as a fundamental basis for conducting genetic research and sexual breeding to a significant extent. Krueger (2002) was the first to report the tetrapolar mating system of *P. umbellatus* and several other species within the genus *Polyporus* in his doctoral dissertation. This report was unique, but which included only a self-crossing chart, it lacked comprehensive information, including details regarding the morphology of the colony [17]. Consequently, more thorough investigations are necessary. Furthermore, advancements in genetics have revealed that mating is regulated by mating-type loci [18]. Two mating-type loci *A* and *B* control the mating in tetrapolar species, and only mating-type locus *A* control mating in bipolar species. The mating-type locus *A* containing a divergently transcribed pair of *HD* (homeodomain) *1* and *HD2* genes, which regulates nuclear pairing, clamp cell formation. and coordinated nuclear division [18,19]. The mating-type locus *B* contains *PR* (pheromone precursor) and *PP* (pheromone receptor) genes [20,21], promoting septal dissolution, nuclear migration towards the apical cell, and clamp cell fusion [18,19]. The increasing availability of genomic information has facilitated the study of mating-type loci, which remain largely unknown for *P. umbellatus*.

The objective of this study was to elucidate the mating system and life cycle of *P. umbellatus* at the molecular level. In this study, the genome of *P. umbellatus* was sequenced and assembled for the first time, and mating-type loci were identified. By integrating data from the mating-type loci with traditional mating tests, the tetrapolar mating system of *P. umbellatus* was elucidated, contributing to a successful case study in the molecular investigation of mating systems. Additionally, the distinct heterothallic life cycle of *P. umbellatus* was characterized. This study provides genetic and molecular evidence regarding the mating system and life cycle of *P. umbellatus*, which may facilitate advancements in genetic research, breeding development, strain improvement, and the industry surrounding *P. umbellatus.*

## 2. Materials and Methods

### 2.1. Fruiting Body Collection and Monokaryotic Offspring Isolation

The fruiting bodies of fungi are fundamental for the investigation of their mating systems. However, the artificial induction of the fruiting body of *P. umbellatus* has not yet been successfully achieved. Therefore, it is essential to collect fruiting bodies from natural habitats to explore the mating system. In this study, fruiting bodies were collected from Changbai Mountain in Jilin Province, which has been designated as Pu-F2 in our previous study [16]. The methodologies for spore print collection, preparation of spore suspension, spreading and collection of single spore isolates (SSIs), as well as the identification of monokaryotic offspring, adhere to the protocols of our earlier study [16]. In this study, one of the verified monokaryotic offspring (SS1) was used to conduct genome sequencing (accession number: JBGTYJ000000000), and totally 19 monokaryotic strains were used to distinguish mating-types.

### 2.2. DNA Preparation, Genome Sequencing, and Assembly

**Figure 1:**
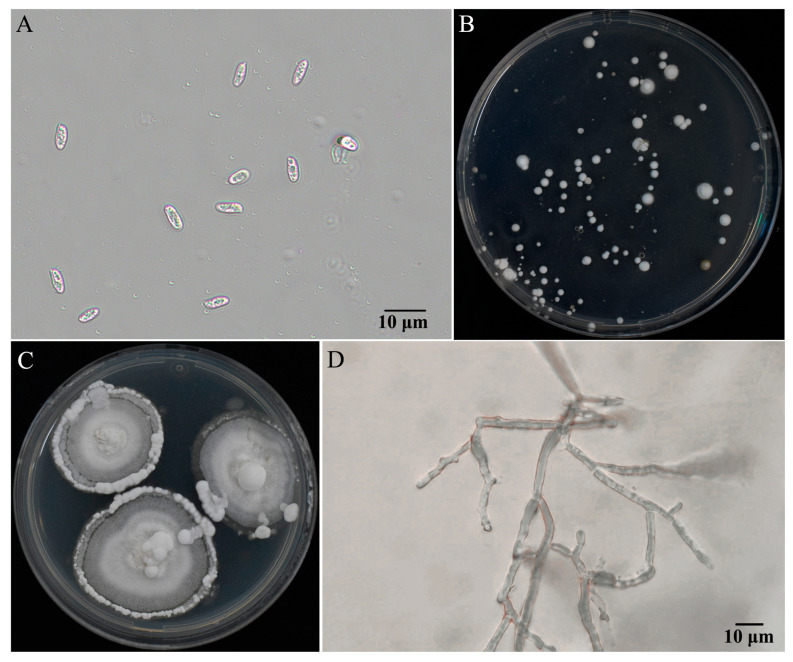
Basidiospores, germination, and monokaryotic strains of Pu-F2. (**A**) Basidiospores of Pu-F2. (**B**) Spore germination after cultured 33 d on PDA media under 25 °C. (**C**) Monokaryotic strain SS1 of Pu-F2 cultured 45 d on PDA media under 25 °C. (**D**) Monokaryotic mycelia of strain SS1.

In contrast to traditional methods that utilize liquid fermentation for sample preparation, the mycelia of *P. umbellatus* exhibited slow growth in liquid media. While the spawn cultured on PDA plates, some clumps of mycelia formed (Figure 1C). These clumps were collected to extract DNA for genome sequencing.

The total genomic DNA was extracted using the conventional CTAB method, and its quality was assessed using the Nanopore One spectrophotometer (NanoDrop Technology, Wilmington, DE, USA) and the Qubit 3.0 Fluorometer (Life Technologies, Carlsbad, CA, USA). The library preparation followed standard protocols for the respective kits. The Illumina NovaSeq 6000 platform and PromethION platforms (Oxford Nanopore Technologies, Oxford, UK) were used for genome sequencing at Wuhan Benagen Tech Solutions Company Limited (Benagen, Wuhan, China).

Raw sequencing data underwent quality assessment and were processed using Oxford Nanopore GUPPY (version 0.3.0) to eliminate unsuccessful reads. The remaining passed reads were used for further analysis. For genome assembly, the software NECAT (https://github.com/xiaochunle/NECAT, accessed on 29 November 2023) was used for preliminary genome assembly, and Racon (version 1.4.11) was utilized for error correction based on ONT sequencing data for two rounds. Further, the software Pilon (version 1.23) was used for error correction based on Illumina sequencing data for two rounds [22]. The Circos graph of the genome was generated using the ‘Advanced Circos’ module of TBtools version 2.086 [23]. The integrity of the genome assembly was estimated using BUSCO (version 4,1,2) [24].

### 2.3. Repeat Annotation, Gene Prediction, and Function Annotation

The combination of ab initio prediction, homology-based prediction, and transcriptome-assisted prediction was used to identify the genes encoding proteins. Combining different prediction strategy can provided a more precise results of coding genes. The software Exonerate (version 2.4.0) was used for homology-based prediction. Augustus (version 3.3.2), Genescan (version 1.0), and GlimmerHMM (version 3.0.4) were employed for ab initio prediction. StringTie (version 2.1.4) and TransDecoder (version 5.1.0) were utilized for transcriptome-assisted prediction. Finally, the software MAKER (version 2.31.10) was used to integrate gene prediction results.

For gene functional annotation, nine databases were utilized, including the UniProt (https://www.uniprot.org (accessed on 29 November 2023)), Pfam (http://pfam.xfam.org/ (accessed on 29 November 2023)), RefSeq (https://www.ncbi.nlm.nih.gov/refseq/ (accessed on 29 November 2023)), Non-Redundant Database (NR, https://ftp.ncbi.nlm.nih.gov (accessed on 29 November 2023)), InterProScan (https://github.com/ebi-pf-team/interproscan (accessed on 29 November 2023)), Clusters of Orthologous Genes (COG, https://www.ncbi.nlm.nih.gov/COG/ (accessed on 29 November 2023)), Kyoto Encyclopedia of Genes and Genomes (KEGG, http://www.genome.jp/kegg/ (accessed on 29 November 2023)), and Pathway and Gene Ontology (GO, http://geneontology.org (accessed on 29 November 2023)) databases. That ensures a comprehensive prediction of gene functions and involved biological processes.

### 2.4. Genome Resequencing

The sample preparation methods are the same as those in Section 2.2. A total of 4 strains, with different mating-types, were subjected to genome resequencing. The genome was sequenced using the high-throughput sequencing instrument DNBSEQ-T7 (BGI, Shenzhen, China). This Whole Genome Shotgun project has been deposited at GenBank under the accession numbers: SRR31745029, SRR31745030, SRR31745027, SRR31745028, and the assembled genomes have been deposited under the accession numbers: JBJZPE000000000, JBJZPF000000000, JBJZPG000000000, JBJZPH000000000.

### 2.5. Mating-Type Identification Based on Mating-Type Genes

According to the annotated mating-type *A* and mating-type *B* loci, four pairs of primers were designed. Two of these primers were developed based on the *HD1* gene of the mating-type *A* locus, while two were designed based on the *PR4* and *PR5* genes of the mating-type *B* locus. The sequences of the primers are as follows:

*HD1*p1F1: 5′-GTGACTAGATCGAACAAAGCTTGC-3′;

*HD1*p1R1: 5′-GTCCAGTTGTCCCGACCTGAATC-3′;

*HD1*p2F2: 5′-GATGGATGATTCTCGACAGTATTCC-3′;

*HD1*p2R2: 5′-CTGAGACCTCAATGCATCTGTTC-3′;

*PR4*F1: 5′-GCCATTGTTGTCTTGATTCCTCTTC-3′;

*PR4*R1: 5′-CGATGCGAGAATAACGTTGAACTG-3′;

*PR5*F1: 5′-CAATCTTTGCTTTCCTTGGTCTC-3′;

*PR5*R1: 5′-CGAGATAGGGACAAGTATAAGCAG-3′.

The genomic DNA of different monokaryotic offspring were extracted from mycelia using a modified CTAB method [25]. The PCR was conducted in a total volume of 25 µL, which included 2 µL of DNA template, 1 µL each of forward and reverse primers, 12.5 µL of 2× mix (Vazyme, Nanjing, China), and 10.5 µL of ddH_2_O. The PCR was conducted with T100^TM^ Thermal Cycler (BIO-RAD, Hercules, CA, USA), and conditions were as follows: an initial denaturation at 95 °C for 5 min, followed by 35 cycles consisting of denaturation at 95 °C for 30 s, annealing at 55 °C for 30 s, and extension at 72 °C for 30 s. The reaction was concluded with a final extension at 72 °C for 10 min and subsequently stored at 4 °C. The amplified products were subjected to separation via electrophoresis on a 1.0% agarose gel. The results of the electrophoresis indicated that the monokaryotic offspring were categorized into distinct mating-types.

### 2.6. Mating Experiments

Mon-to-mon pairing tests were performed among homokaryotic offspring to evaluate mating compatibility. Spawn blocks were inoculated onto PDA plates with a diameter of 60 mm, ensuring a spacing of 1 cm between each block. All plates were incubated at a temperature of 25 °C until the colonies of the various strains made contact with one another. Following this initial contact, the cultures were maintained for several additional days. The formation of clamp connections in the contact zones was examined using a light microscope.

### 2.7. Observation of Hymenium by Scanning Electron Microscope (SEM)

The fruiting bodies of Pu-F2 were fixed using 2.5% glutaraldehyde (McLean Biochemical Technology, Shanghai, China) in a 0.1 M buffer at a temperature of 4 °C for a duration of 24 h. Following fixation, the glutaraldehyde was removed through three washes with deionized water. The samples were then subjected to a dehydration process using increasing concentrations of ethanol: 70%, 85%, 95%, and 100%. Subsequently, the samples were affixed to stubs, coated with a thin conductive layer, and examined using a scanning electron microscope (JEOL, JSM-6510LV, Tokyo, Japan) after undergoing freeze-drying and gold sputtering (JEOL, JFC-1600, Japan).

### 2.8. Nuclear Fluorescent Staining of Hymenium and Basidiospores

The hymenium and basidiospores were collected using sterilized pipette tips from the fruiting body and spore print, and subsequently mixed with 5 μL of a 10 μg/mL solution of 4′,6-diamidino-2-phenylindole (DAPI, Gen-View, El Monte, CA, USA) for a duration of 2 min in a dark environment. The sample was then examined under a fluorescence microscope (Axioimager A2; Zeiss, Göttingen, Germany) utilizing an excitation wavelength of 340 nm and an emission wavelength of 488 nm. The number of nuclei present in basidia and basidiospores were recorded.

## 3. Results

### 3.1. Basidiospore Isolation and Monokaryotic Offspring Identification

The basidiospores of *P. umbellatus* are cylindical (ratio of length/width, Q > 2.0, Figure 1A), which were germinated to visible colony after 12 d. The germination ratio of basidiospores were 30.2%, 25.7%, and 29.1% for three replicates (Figure 1B). Colonies of monokaryotics strains (Figure 1C) were similar to those of the dikaryotic strain [16], while the mycelia of monokaryotic strains had no clamp connections (Figure 1D). More than 30 monokaryotic offspring of Pu-F2 were collected in this study.

### 3.2. Genome Sequencing, Assembly, and Annotation of P. umbellatus

**Figure 2:**
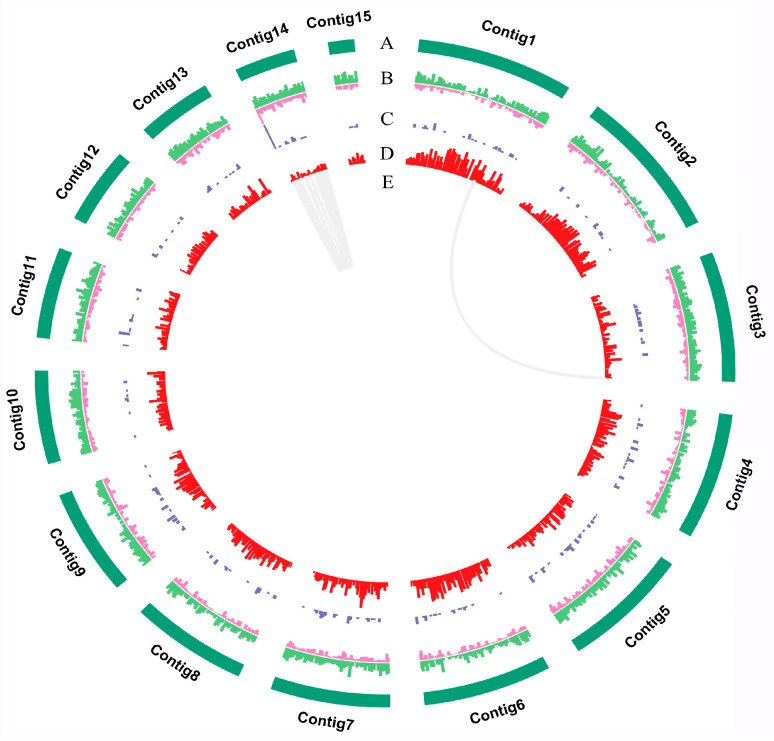
Circos graph of genome characteristics of *Polyporus umbellatus*. (**A**) 14 contigs; (**B**) repeat sequences of which transposons are in green and tandem repeat in pink; (**C**) non-coding RNA (ncRNA); (**D**) gene density; (**E**) large fragment duplication.

A de novo genome assembly was performed by integrating Nanopore long-read sequencing and Illumina short-read sequencing (Figure 2). A genome of 71.68 Mb was assembled based on 7,119,902 Nanopore reads (~150× coverage, 10.59 GB data size), which consists of 27 contigs with a contig N50 ~5.02 MB, with the longest contig being 6.57 MB (Table 1). A large amount of original sequencing data ensures the integrity of genomic data, and the length of the contig N50 indicates a high assembly quality. The genome size of *P. umbellatus* is relatively large in the Polyporales, being smaller only than *Postia placenta* (90.93 Mb), *Porosteum spadiceum* (85.20 Mb), *Perenniporia subacida* (81.17 Mb), and *Ganoderma boninense* (79.19 Mb). It is also larger than the sclerotia-formation fungus *Wolfiporia hoelen* (Chinese Fuling, 64.44 Mb, [26]) (Supplementary Table S1). The mapping rate of Illumina data is 99.86%, the genome coverage is 99.97%, and 97.2% of complete BUSCOs all indicate the good genome integrity of *P. umbellatus* (Supplementary Tables S2 and S3).

A large number of repetitive sequences were annotated in *P. umbellatus* strain PuF2-SS1, accounting for 61.95% of the whole genome. Among these, 44.66% are transposons, 1.78% are tandem repeats, and 15.51% are unknown repetitive sequences. For transposons, 7.72% are Class II (transposon) and 36.94% are Class I (retrotransposon). Retrotransposons consisted of 32.62% long terminal repeats (LTRs), 4.21% long interspersed nuclear elements (LINEs), and 0.06% short interspersed nuclear elements (SINEs). The most abundant LTRs were Gypsy, accounting for 25.16% of the genome. Transposons only account for 7.72% of DNA transposons. Approximately 1.78% of the genome was identified as tandem repeats, with a total of 1231 SSRs identified (Table 2). The ratio of repetitive sequences of *P. umbellatus* was more than that of *W. hoelen* (48.56%) which have high ratio repetitive sequences among different species [26].

A total of 9574 genes were predicted based on de novo prediction, homology prediction, and RNA–seq prediction, with an average sequence length of 2715.65 bp. Each predicted gene contained 6.87 exons with an average length of 313.36 bp and 5.87 introns with an average length of 96.14 bp (Supplementary Table S4). 97.3% complete BUSCOs of gene prediction indicate the high annotation quality of *P. umbellatus* (Supplementary Table S5). To obtain comprehensive gene function information, all 9574 genes were annotated in various databases. Most of these genes were mapped using the Nr database, followed by Uniprot, InterProScan, RefSeq, GO, Pfam, KEGG, Pathway, KOG, and TigerFam (Table 1). For the non-coding genes, 766 ncRNAs were predicted, including 66 rRNAs, 21 snRNAs, 678 tRNAs, and 1 miRNA, which collectively occupied 0.198% of the genome (Supplementary Table S6).

### 3.3. Mating Loci of P. umbellatus

**Figure 3:**
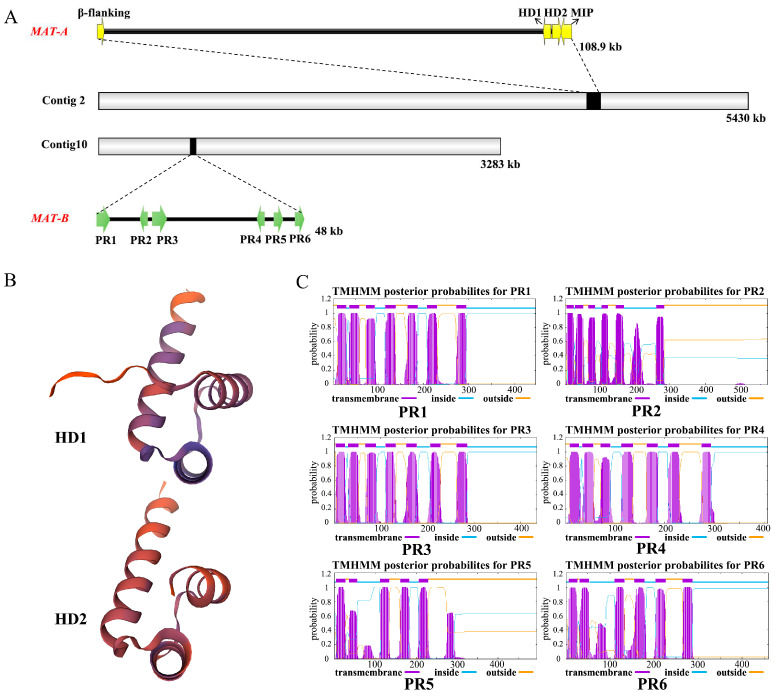
Mating-type loci of *P. umbellatus*. (**A**) *MAT-A* and *MAT-B* loci. (**B**) Protein three-dimension structure of HD1 and HD2 proteins predicted using SWISS-MODEL. (**C**) Trans-membrane-spanning domain of six PR proteins predicted using TMHHM.

The initial genome analysis of the monokaryotic strain Pu-F2-SS1 of *P. umbellatus* revealed the mating-type *A* (*MAT-A*) and *B* (*MAT-B*) loci, which were located on Contig 2 and Contig 10, respectively (Figure 3A). In basidiomycetes, the mating-type *A* and *B* loci are typically situated on different chromosomes in species exhibiting a tetrapolar mating system, indicating that these loci are unlinked. This observation is consistent with the findings for *P. umbellatus*, as examined in this study.

#### 3.3.1. Mating-Type Locus A

**Figure 4:**
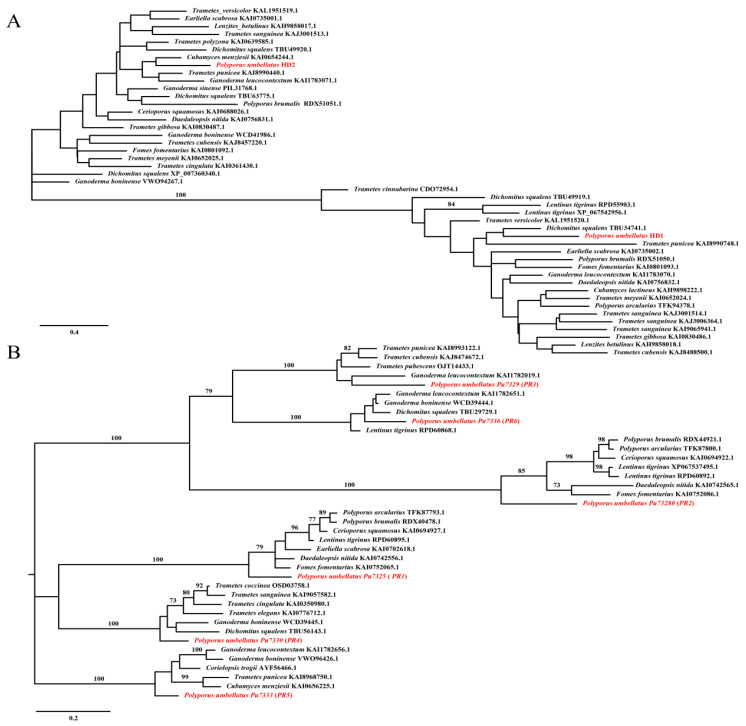
Phylogenetic analysis based on protein sequences of mating-type genes using PlyloSuite. (**A**). IQ-TREE of HD genes. (**B**). IQ-TREE of PR genes.

The mating-type locus *A* contains a pair of *HD* (homeodomain) genes that exhibit different coding orientations. Additionally, the *β-fg* (*β-flanking*) and *MIP* genes are located upstream and downstream of the *HD1-HD2* pair, respectively, as annotated in this study. The *MIP* gene is situated in close proximity to *HD2* and exhibits a “head-to-head” coding direction. In contrast, the *β-fg* gene is positioned upstream of *HD1*, approximately 110 kb away, and has a reverse coding direction (Figure 3A). Notably, there are numerous repetitive sequences between the *β-fg* region and *HD1*, which is consistent with findings in *W. hoelen* [26]. The *HD1* gene is 1374 bp in length, while *HD2* is 1413 bp long; the corresponding proteins for HD1 and HD2 consist of 457 and 470 aa, and which contain three and four introns separately (Supplementary Figure S1). The three-dimensional structures of the HD1 and HD2 proteins were constructed using SWISS-MODEL, revealing that both proteins possess the conserved structural features characteristic of homeodomain (Figure 3B). In the phylogenetic tree, the HD1 and HD2 cluster on different branches with high bootstrap value, which shows the genes *HD1* and HD2 were formed prior to the species formation of *P. umbellatus*. Meanwhile, the *HD* genes exhibit similarities to species within the Polyporales, suggesting that the evolution of *HD* genes is closely correlated with phylogenetic relationships (Figure 4A).

#### 3.3.2. Mating-Type Locus B

In the genome of *P. umbellatus*, six *PR* (pheromone receptor) genes (*PR1*-*PR6*) (*Pu7325*, *Pu7328*, *Pu7329*, *Pu7330*, *Pu7333*, *Pu7336*) have been annotated (Figure 3A). The *MAT-B* loci span 48 kb from *PR1* to *PR6*. The lengths of the *PR* genes range from 1505 bp to 1846 bp, with corresponding protein lengths varying from 407 aa to 579 aa (Supplementary Table S7). No pheromone precursors were identified through open reading frame (ORF) prediction and conserved motif searches in the upstream and downstream regions of the *PR* genes. Among the six *PR* genes, *PR2* is located in proximity to *PR3*, *PR4* is located in proximity to *PR5* and *PR6*, while *PR1* is situated separately. Different PR genes have distinct gene structure, the exons varies from three to six (Supplementary Figure S2). All six *PR* genes possess seven possible transmembrane-spanning domains, and three of which high accuracy as predicted by TMHMM (https://services.healthtech.dtu.dk/services/TMHMM-2.0/, accessed on 5 September 2024) (Figure 3C), which serves as an identifying characteristic. Obviously, the proteins are not highly conserved, only 11 amino acids are consistently conserved among the six *PR* genes of *P. umbellatus* (Supplementary Figure S3). In the phylogenetic tree, the six *PR* genes cluster into distinct branches with high bootstrap values, indicating that these different *PR* genes were formed prior to the species formation of *P. umbellatus*. Furthermore, all *PR* genes exhibit similarities to species within the Polyporales, suggesting that the evolution of *PR* genes is closely correlated with phylogenetic relationships (Figure 4B).

### 3.4. Mating-Type Discrimination Based on Mating-Type Loci

**Figure 5:**
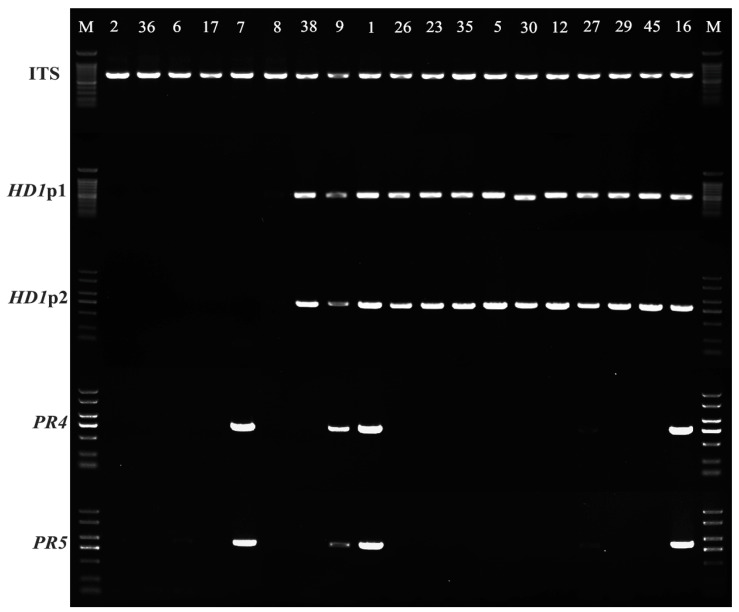
Identification of mating-types based on the analysis of mating-type loci. Strains that successfully amplified the genes *HD1* and *PR* were designated as *A1B1*. In contrast, only the gene *HD1* was successfully amplified and designated as *A1B2*, while only the gene *PR* was successfully amplified and designated as *A2B1*. Notably, both genes *HD1* and *PR* failed to amplify the designation *A2B2*. The “SS” was omitted in the figure.

The PCR products of genomic DNA of different monokaryotic offspring were analyzed using electrophoresis on an agarose gel (Figure 5). The clear amplified bands of ITS represents the high-quality of DNA templates, and high similarity of electropherograms obtained between the two primers designed for *HD1*, as well as between those for *PR4* and *PR5*, demonstrate the reliability of the results. Based on the electrophoresis findings, the monokaryotic strains were classified into four distinct types (Figure 5), which present the tetrapolar mating system of *P. umbellatus*. Given that the *HD* genes are located on the mating *A* loci and the *PR* genes are situated on the mating *B* loci, the similar electropherograms of *HD* indicate the same mating-type *A*, while the analogous electropherograms of *PR* indicate the same mating-type *B*. Strains SS9 and SS16 exhibited similarities to strain SS1, which was the genome-sequenced strain in this study, and were designated as mating-type *A1B1*. The mating-type of strains SS2, SS6, SS8, SS17, and SS36 were classified as *A2B2*, while strain SS7 was designated as *A2B1*. Additionally, the mating-types of strains SS38, SS26, SS23, SS35, SS5, SS30, SS12, SS27, SS29, and SS45 were classified as *A1B2*.

### 3.5. Mating Verification of Mating-Types

**Figure 6:**
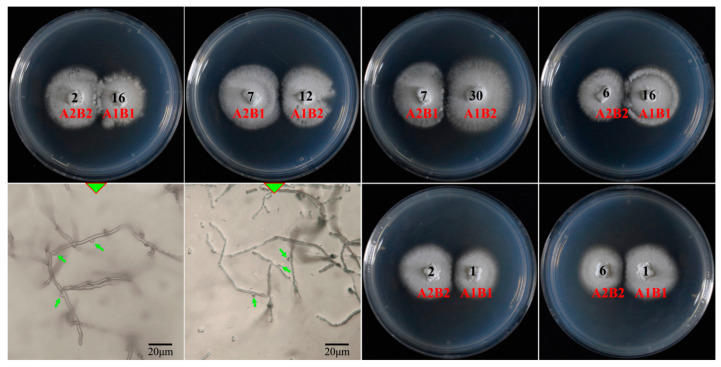
The mating verification of the monokaryotic strains exhibiting different mating-types. The Petri plates demonstrated successful mating across all six pairs of combinations. Microscopic analysis revealed the successful mating (clamp connection formation) between strains SS2 and SS16, as well as between strains SS7 and SS12. The “SS” was omitted in the figure.

Based on the results obtained from the analysis of mating-type loci, a total of seven strains were selected for mating tests. These included strains SS1 and SS16, which possess mating-type *A1B1*; strains SS2 and SS6, which exhibit mating-type *A2B2*; strains SS12 and SS30, which carry mating-type *A1B2*; and strain SS7, which has mating-type *A2B1*. The outcomes of the mating tests were consistent with the previously identified mating-types. In the mono-to-mono pairing tests involving the selected seven strains, strains SS1 and SS16 were able to mate with strains SS2 and SS6, while strain SS7 successfully mated with strains SS12 and SS30 (Figure 6). The formation of clamp connections served as an indicator of successful mating (Figure 6). Conversely, visible reactions between colonies provided limited evidence of mating. For instance, the contact reaction observed between strains SS2 and SS1 was similar to that between strains SS2 and SS30, despite the fact that these pairings resulted in successful and unsuccessful mating, respectively (Supplementary Figure S4). Additionally, the contact reaction between strains SS2 and SS16 bore similarities to that between strains SS12 and SS16 (Supplementary Figure S4).

### 3.6. Discrepancy Between Mating-Type Loci A1 and A2, and B1 and B2

**Figure 7:**
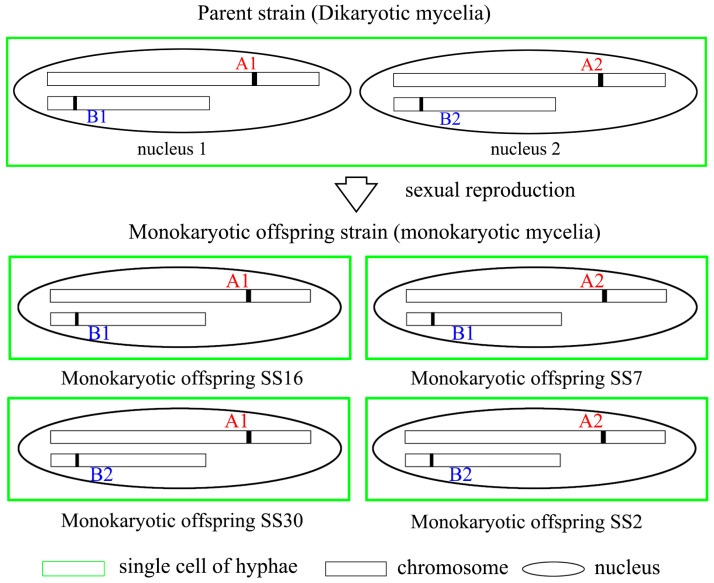
The distribution of mating loci in parent strain and monokaryotic offspring. A1, A2 indicate different mating type *A* loci, B1, B2 indicate different mating type *B* loci.

Four monokaryotic strains, specifically strains SS2, SS7, SS16, and SS30, were selected for genome resequencing due to their possession of distinct mating loci. Strain SS16 shares the *A1* mating locus with strain SS30, while strain SS2 shares the *A2* locus with strain SS7. Additionally, strain SS16 shares the *B1* locus with strain SS7, and strain SS2 shares the *B2* locus with strain SS30 (Figure 7). In total, the sequencing yielded 4.0 Gb (approximately 56×), 3.2 Gb (approximately 44×), 4.0 Gb (approximately 56×), and 4.9 Gb (approximately 63×) data for strains SS2, SS7, SS16, and SS30, respectively. The genomes were subsequently assembled to sizes of 72.0 Mb, 72.5 Mb, 71.8 Mb, and 77.2 Mb for strains SS2, SS7, SS16, and SS30, respectively. The corresponding genes for *HD* and *PR* were annotated using Softberry (http://www.softberry.com/, accessed on 5 September 2024) and BLAST.

A comparative analysis of the mating loci *A1* and *A2* reveals significant sequence divergence between these loci. The nucleotide identity between *A1* and *A2* is only 34.82% and 45.79% for *HD1* and *HD2*, respectively, while the protein identity is markedly lower, at 8.95% and 17.32% for HD1 and HD2 (Supplementary Figure S5). That presents the *HD1* and *HD2* codetermined the mating *A* types. In contrast to the mating loci *A*, only half of the *PR* genes exhibit substantial sequence divergence. Specifically, *PR1*, *PR2*, and *PR3* demonstrate minimal variation, with nucleotide identities of 99.94%, 99.78%, and 99.93%, respectively, and a protein identity of 100% for all three genes. Conversely, *PR4*, *PR5*, and *PR6* show considerable differences between *B1* and *B2*, with nucleotide identities of 35.01%, 39.29%, and 41.43%, and protein identities of 27.54%, 27.72%, and 29.33%, respectively (Supplementary Figure S6). That presents the *PR4*, *PR5* and *PR6* codetermined the mating *B* types. Which in accordance with the results, the primers designed on *HD1* and *PR4*, *PR5* can distinguish monokaryotic strains with different mating-types.

### 3.7. The Number of Sterigma and Basidiospores Observed by SEM

**Figure 8:**
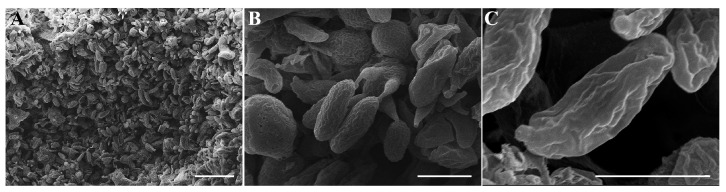
Hymenium morphology observed by SEM. (**A**) Image of hymenium magnified 800 times. (**B**) Four-sterigma basidium with four basidiospores. (**C**) Basidiospores. Bar = 5 μm.

The quantity of basidiospores and sterigma per basidium significantly influences the life cycle of fungi. To facilitate a comprehensive examination of the hymenium structure, the magnification was gradually increased (Figure 8). Based on the results obtained from SEM observations of the hymenium, only four-sterigma basidia were identified (Figure 8A), with each basidium producing four basidiospores (Figure 8B). This finding is consistent with the characteristics observed in the majority of common mushroom species.

### 3.8. Nuclear Behaviors of Basidia and Basidiospores

**Figure 9:**
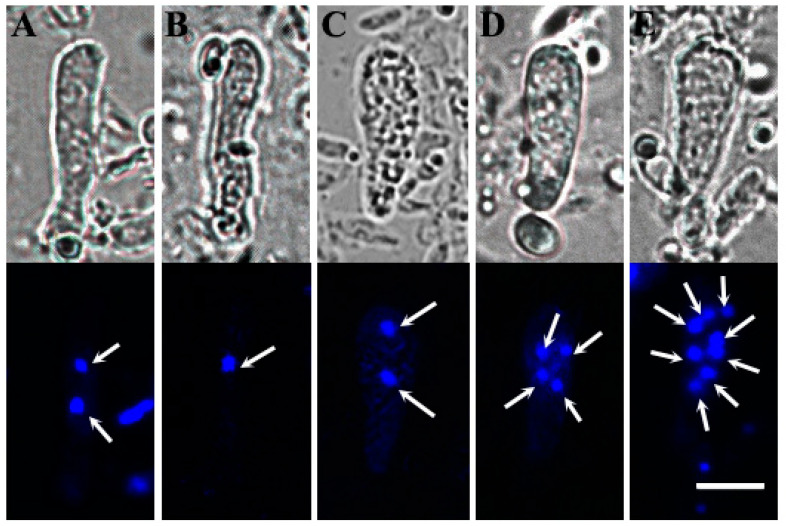
Nuclear behaviors of basidia at different developmental stages. (**A**) Binucleate phase of young basidium, (**B**) karyogamy, (**C**) meiosis I, (**D**) meiosis II, (**E**) post-meiotic mitosis. Arrows indicate the nuclei. Bar = 5 μm.

The fragments of fruiting bodies were stained with DAPI, and the nuclear behavior was examined (Figure 9). Similar to other basidiomycetes, the initial two-nuclei stage was observed (Figure 9A), followed by the karyogamy process, during which fused nuclei were formed (Figure 9B). Meiosis subsequently occurred after karyogamy, resulting in the formation of two nuclei after meiosis I (Figure 9C), and four nuclei after meiosis II (Figure 9D). Furthermore, the process did not cease at this point; basidia containing eight nuclei were observed (Figure 9E), indicating that post-meiotic mitosis had taken place.

**Figure 10:**
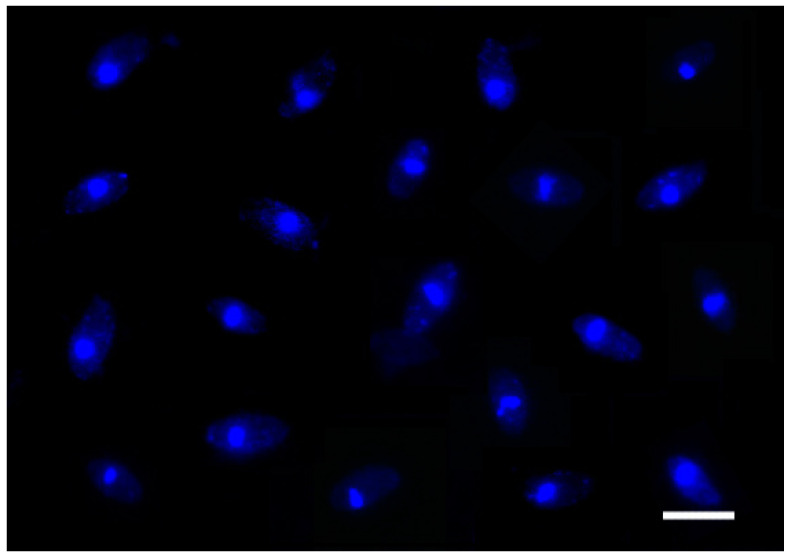
Nuclear staining of basidiospores with DAPI. Bar = 5 μm.

The number of nuclei in basidiospores significantly influences the complexity of the life cycle. In *P. umbellatus*, only uninucleate basidiospores were observed (Figure 10), indicating that each basidiospore possesses a single mating-type. Consequently, *P. umbellatus* exhibits a relatively simple heterothallic life cycle.

## 4. Discussion

The cultivation of *P. umbellatus* is predominantly reliant on sclerotia through asexual reproduction, which has led to significant homogenization and degeneration of seed sclerotia. In contrast, sexual reproduction has proven to be an effective strategy for addressing these challenges, with a clear mating system serving as the foundation for implementing sexual breeding. This study comprehensively elucidates the tetrapolar mating system of *P. umbellatus* by integrating mating-type loci analysis derived from genomic data with empirical mating tests. Furthermore, the heterothallic life cycle of this species is also characterized. These findings are expected to advance both the genetic research and breeding practices associated with *P. umbellatus*.

### 4.1. Study Methods of Mating System

The mating system serves as a fundamental basis for conducting genetic studies and breeding programs. Consequently, the investigation of mating systems has gained prominence in the context of edible and medicinal mushrooms. Notably, certain species exhibit unique sexual life cycles, which enhance the appeal of such studies; examples include *Agaricus bisporus* [27], *Volvariella volvaceae* [28], and *W. hoelen* [29]. The traditional approach to studying mating systems has involved a three-round mating test, which has been employed for the majority of species [30,31,32,33,34]. However, advancements in genomics and molecular technologies have led to the emergence of more effective methodologies, including the differentiation of monokaryotic and homokaryotic strains [35,36]. In previous research, a method for distinguishing homokaryotic strains of *W. hoelen* based on single nucleotide polymorphisms (SNPs) in the *rpb2* gene was developed, representing a critical component of the mating system study for this species [29].

The mating-type loci have been identified as key regulators of mating in mono- or homokaryotic strains possessing compatible mating-type loci. Consequently, these loci would become the primary focus of research, while mating tests have served as supplementary methods. However, some studies have opted to forgo mating tests altogether. In the present study, both mating tests and mating-type loci analyses were conducted, which confirmed the existence of a tetrapolar mating system in *P. umbellatus*. With the reduction in sequencing costs, the genomes of various species have been published [37,38,39,40], and the analysis of mating-type loci has become a standard component of genomic studies [41,42]. This development presents a unique opportunity to investigate mating systems, and it is anticipated that numerous species will have their mating systems elucidated as their genomes are sequenced.

### 4.2. Nuclear Distribution and Migration Model in Basidia and Basidiospores

Nuclear distribution and migration present a complex and intriguing area of study, characterized by significant observational challenges [43,44]. Previous research has identified and categorized the nuclear distribution models across various species into six distinct types. Type I illustrates the formation of eight nuclei within the basidia through the processes of meiosis and subsequent post-meiotic mitosis, leading to the development of eight unicellular basidiospores. Type II describes a scenario in which post-meiotic mitosis occurs within the sterigma, resulting in the allocation of four nuclei to four basidiospores, thereby producing four unicellular basidiospores, while the remaining four nuclei revert to the basidia. Type III indicates that post-meiotic mitosis takes place within four basidiospores, with one nucleus returning to the basidia for each basidiospore, ultimately yielding four unicellular basidiospores. Type IV depicts a situation where post-meiotic mitosis occurs within four basidiospores, culminating in the formation of four binucleate basidiospores. Type V outlines the formation of four nuclei post-meiosis, which are then allocated to four basidiospores, resulting in four unicellular basidiospores. Finally, Type VI describes the formation of eight nuclei following post-meiotic mitosis within the basidia, which are subsequently transferred to form four binucleate basidiospores [45]. In addition to these six models, other variations exist. For instance, in *A. bisporus*, only two basidiospores are formed from a single basidium, with four nuclei being allocated to these two basidiospores, resulting in the formation of two binucleate basidiospores [46]. The current study observes the formation of eight nuclei within the basidia, with only four nuclei being transferred to produce four unicellular basidiospores, suggesting the emergence of a novel model. Thus, there are at least eight distinct models of nuclear distribution and migration in the basidia and basidiospores of basidiomycetes.

### 4.3. Heterothallic Life Cycle of Polyporus umbellatus

**Figure 11:**
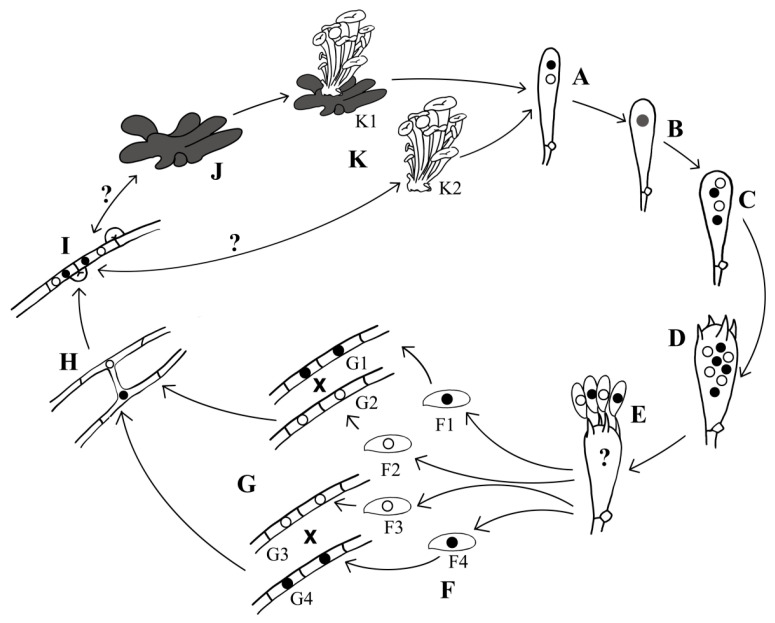
Heterothallic life cycle of *Polyporus umbellatus*. (**A**) Binucleate phase of young basidium, (**B**) karyogamy, (**C**) meiosis, (**D**) post-meiotic mitosis, (**E**) formation of basidiospores, (**F**) basidiospores (F1. ininucleate spore with mating-type *A1B1*. F2. uninucleate spore with mating-type *A2B2*. F3. uninucleate spore with mating-type *A1B2*. F4. uninucleate spore with mating-type *A2B1*), (**G**) monokaryotic hypha (G1. monokaryotic hypha with mating-type *A1B1*. G2. monokaryotic hypha with mating-type *A2B2*. G3. monokaryotic hypha with mating-type *A1B2*. G4. monokaryotic hypha with mating-type *A2B1*), (**H**) plasmogamy, (**I**) dikaryotic hypha, (**J**) sclerotia, (**K**) fruiting bodies (K1. fruiting bodies formed on sclerotia. K2. fruiting bodies formed on artificial media). Question mark indicates that there is no evidence.

According to the findings of this study, the sexual life cycle of *P. umbellatus* has been summarized (Figure 11). The fruiting body represents the sexual stage (Figure 11K), while the hymenium functions as the sexual “organ”, basidia are formed, where karyogamy occurs (Figure 11A,B), followed by meiosis (Figure 11B,C) and post-meiotic mitosis (Figure 11C,D). Subsequently, the nuclei are transferred from the basidia to the basidiospores, resulting in the formation of four basidiospores per basidium (Figure 11E), with each basidium containing a single nucleus (Figure 11F). Four distinct basidiospore types are produced (Figure 11F1–F4), each exhibiting different mating-types: *A1B1*, *A2B2*, *A1B2*, and *A2B1*. Following this, the basidiospores germinate into monokaryotic hyphae (Figure 11G). Hyphae that carry compatible mating-type loci are capable of mating (Figure 11H), leading to the formation of dikaryotic hyphae (Figure 11I). Typically, the dikaryotic mycelia can directly develop into sclerotia (Figure 11J) and fruiting bodies (Figure 11K2), with fruiting bodies also capable of arising from sclerotia (Figure 11K1).

While there are some steps in the process remain ambiguous, such as the nuclear migration model from basidia to basidiospores, the formation of dikaryotic mycelia into sclerotia and fruiting bodies has not yet been achieved. Consequently, we have to collect fruiting bodies from the field. Previous research has extensively investigated the induction of sclerotia and fruiting bodies using pure spawn. Notably, Pasailiuk (2020) was the first to report on the artificial cultivation of fruiting bodies of *P. umbellatus*, marking a unique contribution to the field [12]. In China, numerous attempts have been made to cultivate sclerotia [47,48,49,50]; however, only minimal sclerotia-shaped tissue has been produced, and there has yet to be a successful application of pure spawn for the cultivation of sclerotia of *P. umbellatus*. Given that sclerotia and fruiting bodies represent the medicinal and edible components of this species, further research in this area is warranted.

### 4.4. Mating-Type Loci Structure

The species exhibiting a tetrapolar mating system, governed by unlinked mating-type *A* and *B* loci, includes *P. umbellatus*. This species has been shown to possess a tetrapolar mating system, with the mating-type *A* loci located on Contig 2 and the mating-type *B* loci situated on Contig 10. To date, the genome of *P. umbellatus* represents the first complete genome sequenced for the genus *Polyporus*, as *P. brumalis*, *P. arcularius*, and *P. squamosus* have been reclassified into other genera based on taxonomic revisions [51,52]. The structure of mating loci appears to be relatively conserved among basidiomycetes [53]. In comparison to other species within the order Polyporales, the mating-type loci of *Grifola frondosa* have recently been characterized [30]. This species contains one pair of *HD* genes and six *PR* genes, exhibiting structural similarities to *P. umbellatus*. Additionally, both *W. hoelen* and *W. cocos* possess three *HD* genes and four *PR* genes, while *Laetiporus sulphureus* has four *HD* genes and two *PR* genes [25]. All these species exhibit one pair of *HD* genes oriented in opposite directions, along with additional *HD* genes. Specifically, the third *HD* gene is located at a considerable distance from the *HD1-HD2* pair downstream of the *MIP* in *W. hoelen* and *W. cocos*, whereas the additional *HD* genes in *L. sulphureus* are situated between the *MIP* and *HD2* [25]. In *Sparassis latifolia* and *S. crispa*, one pair of *HD* genes and six *PR* genes have been annotated. The *MIP* and *β-fg* genes are conserved, consistently located adjacent to the *HD1-HD2* pair, although their orientation varies [54]. In the species *G. frondosa*, *S. latifolia*, and *W. hoelen*, the *MIP* gene is positioned on the side of *HD2*, with the *β-fg* gene on the side of *HD1*. Conversely, in *Laccaria bicolor* and *Pleurotus djamor*, the orientation is reversed [30]. The number of *HD* and *PR* genes, as well as the structure of mating loci, has undergone extensive evolutionary changes. A comprehensive analysis will elucidate the evolutionary processes based on genomic information from an increasing number of species.

## 5. Conclusions

Our study presents the inaugural sequencing and assembly of the genome of *P. umbellatus* utilizing both Nanopore and Illumina sequencing platforms. Through the annotation of mating-type loci, the differentiation of monokaryotic offspring exhibiting various mating-types, and the execution of pairing tests, we elucidated the tetrapolar mating system of *P. umbellatus*. Additionally, by integrating the resequencing of monokaryotic strains with distinct mating-types, we identified that *HD1* and *HD2* correspond to mating-type *A*, while *PR4*, *PR5*, and *PR6* correspond to mating-type *B*. Ultimately, the heterothallic life cycle was characterized through nuclear fluorescent staining and scanning electron microscopy. This study not only advances the breeding and genetic research of *P. umbellatus*, but also serves as a successful case study in the molecular investigation of mating systems.

## Figures and Tables

**Table 1:** *P. umbellatus* genome assembly and functional annotation.

Item	Value	Item	Number	Percentage
Total_length (bp)	71,684,542	All	9574	100%
Contigs	27	Nr	8617	90%
GC_content(%)	48.8	Uniprot	8608	89.91%
N50 (bp)	5,024,974	Interpro	8496	88.74%
N90 (bp)	3,210,685	Refseq	8395	87.69%
Average (bp)	2,654,983	GO	6044	63.13%
Median (bp)	2,592,259	KEGG	3429	35.82%
Min (bp)	22,957	Pathway	1888	19.72%
Max (bp)	6,571,211	KOG	1807	18.87%
Total number of genes	9574	Tigerfam	1778	18.57%

**Table 2:** Classification of the repeat sequences in the genome of *P. umbellatus*.

Class	Order	Superfamily	Repeat_Type_Size (bp)	Ratio (%)
ClassI	LTR	Gypsy	18,033,317	25.16
		Copia	5,169,664	7.21
		other	175,494	0.25
	LINE	Tad1	2,499,759	3.49
		other	555,553	0.77
	SINE	SINE	44,728	0.06
ClassII	DNA		5,549,287	7.72
Total TEs			31,852,308	44.66
Tandem repeats	Satellite		1,273,992	1.78
Unknown			11,114,783	15.51
Total repeats				61.95

## Data Availability

The data presented in this study are available in the Supplementary Figures and Tables. The genome of *Polyporus umbellatus* has been submitted to NCBI with BioProject: PRJNA1153740, accession numbers: JBGTYJ000000000, JBJZPE000000000, JBJZPF000000000, JBJZPG000000000, JBJZPH000000000, SRR31745029, SRR31745030, SRR31745027, SRR31745028.

## Supplementary Materials

The following supporting information can be downloaded at: https://www.mdpi.com/article/10.3390/jof11010015/s1. Figure S1: Gene structure of *HD* genes of mating-type locus *A*; Figure S2: Gene structure of *PR* genes of mating-type locus *B*; Figure S3: Protein sequence alignment of six *PR* genes; Figure S4: Mon-to-Mon pairing tests; Figure S5: Alignment of *HD* genes of mating loci *A1* and *A2*; Figure S6: Alignment of *PR* genes of mating loci *B1* and *B2*; Table S1: Genome size of different species in Polyporales; Table S2: Statistics of illumina data mapping of *P. umbellatus* genome; Table S3: Statistics of BUSCO evaluation of *P. umbellatus* genome; Table S4: Annotation statistics of *P. umbellatus* coding genes; Table S5: Statistics of BUSCO evaluation of *P. umbellatus* gene prediction; Table S6: Statistics of non-coding RNA annotation results in *P. umbellatus*; Table S7: Length of *HD* and *PR* genes in *P. umbellatus*.
