# Supplementary material for: Genome Sequencing Providing Molecular Evidence of Tetrapolar Mating System and Heterothallic Life Cycle for Edible and Medicinal Mushroom Polyporus umbellatus Fr
Source: J Fungi (Basel). 2024 Dec 28;11(1):15. doi: 10.3390/jof11010015 (PMC11766841; doi:10.3390/jof11010015)
Supplement: Supplementary file 1 [file jof-11-00015-s001.zip › jof-3332177-Supplementary Figures.pdf]

Genome Sequencing Providing Molecular Evidence of Tetrapolar Mating System and Heterothallic Life Cycle for Edible and Medicinal Mushroom *Polyporus umbellatus* Fr.

Shoujian Li [1,2], Youyan Liu [1,2], Liu Liu [1,2], Bing Li [1,2] and Shunxing Guo [1,2,*]

[1] The Institute of Medicinal Plant Development, Chinese Academy of Medical Sciences & Peking Union Medical College, Beijing 100193, China; 13756298450@163.com(S.L.); y1095192105@163.com (Y.L.); liuliu0026@foxmail.com (L.L.); zudengtianxia@126.com (B.L.);

[2] State Key Laboratory of Bioactive Substance and Function of Natural Medicines, Chinese Academy of Medical Sciences and Peking Union Medical College, Beijing 100193, China

* Correspondence: sxguo1986@163.com; Tel.: +86-010-57833231

Supplementary Figures

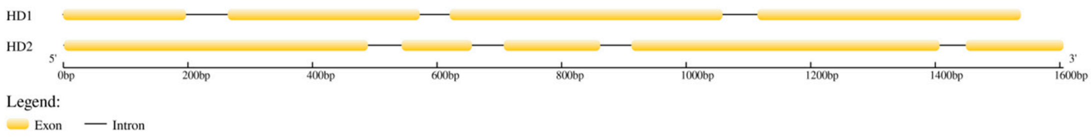

Figure S1: Gene structure of *HD* genes of mating-type locus *A*.

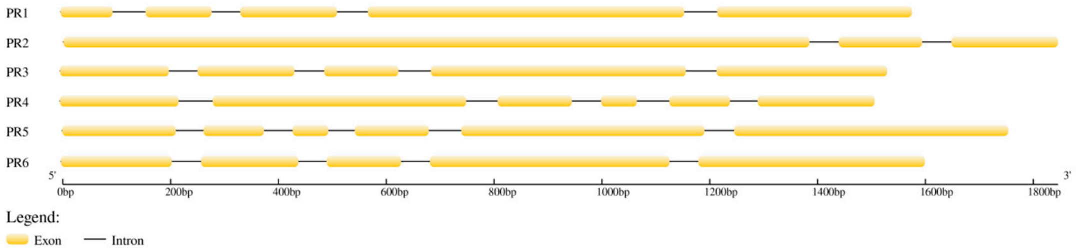

Figure S2: Gene structure of *PR* genes of mating-type locus *B*.

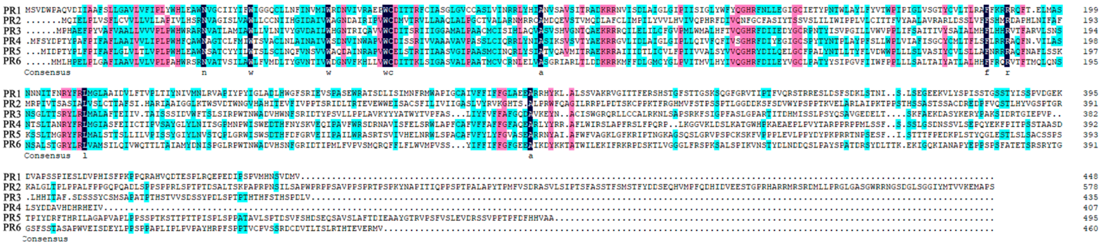

Figure S3: Protein sequence alignment of six *PR* genes.

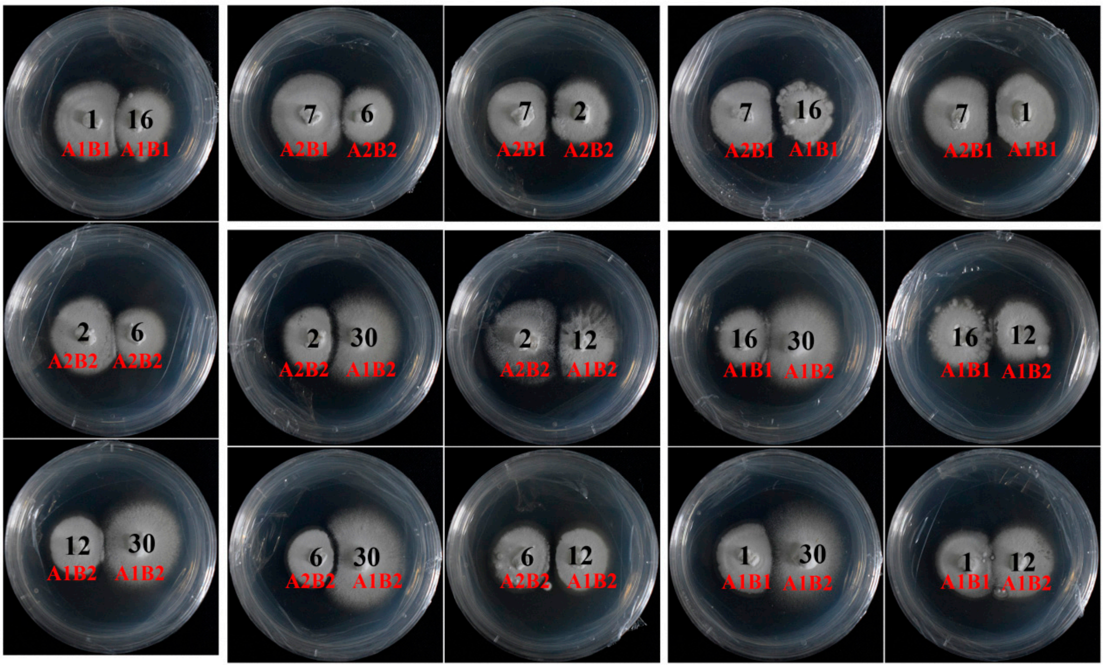

Figure S4: Mon-to-Mon pairing tests.

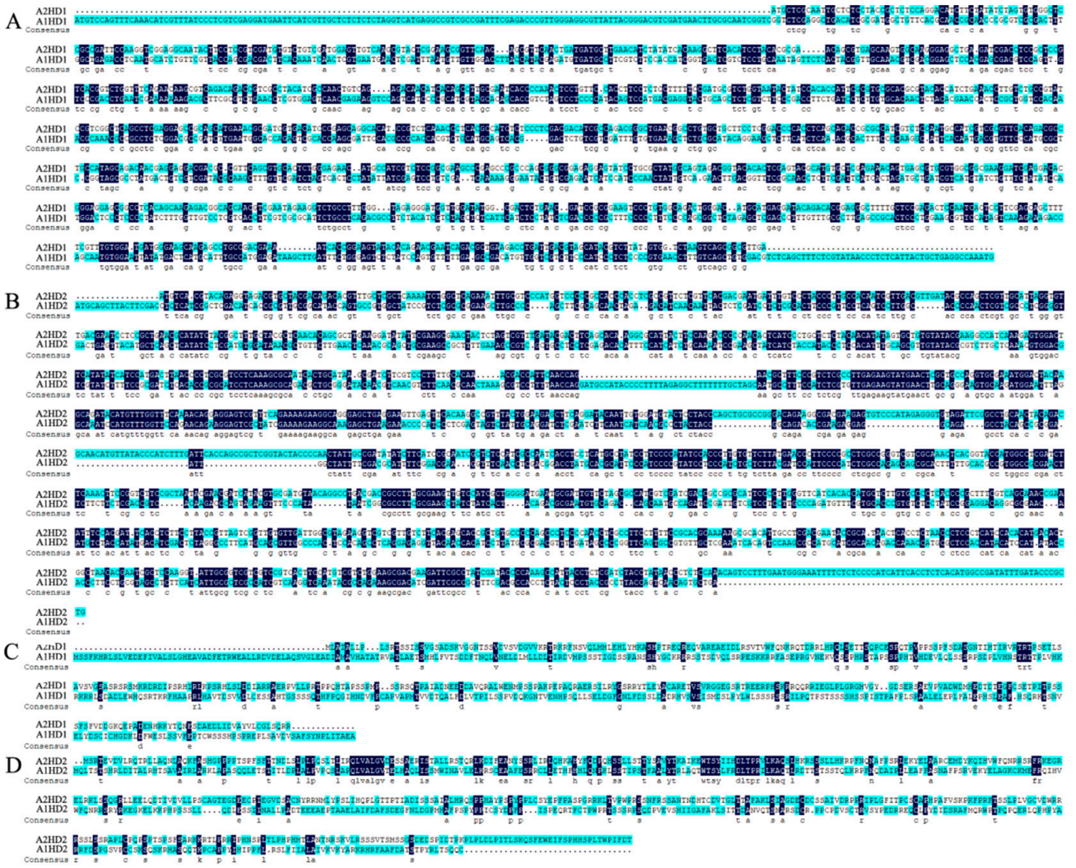

Figure S5: Alignment of *HD* genes of mating loci *A1* and A2. (**A**) DNA sequence alignment of *HD1* of mating loci *A1* and *A2*. (**B**) DNA sequence alignment of *HD2* of mating loci *A1* and *A2*.(**C**) Protein sequence alignment of *HD1* of mating loci *A1* and *A2*. (**D**) Protein sequence alignment of *HD2* of mating loci *A1* and *A2*.

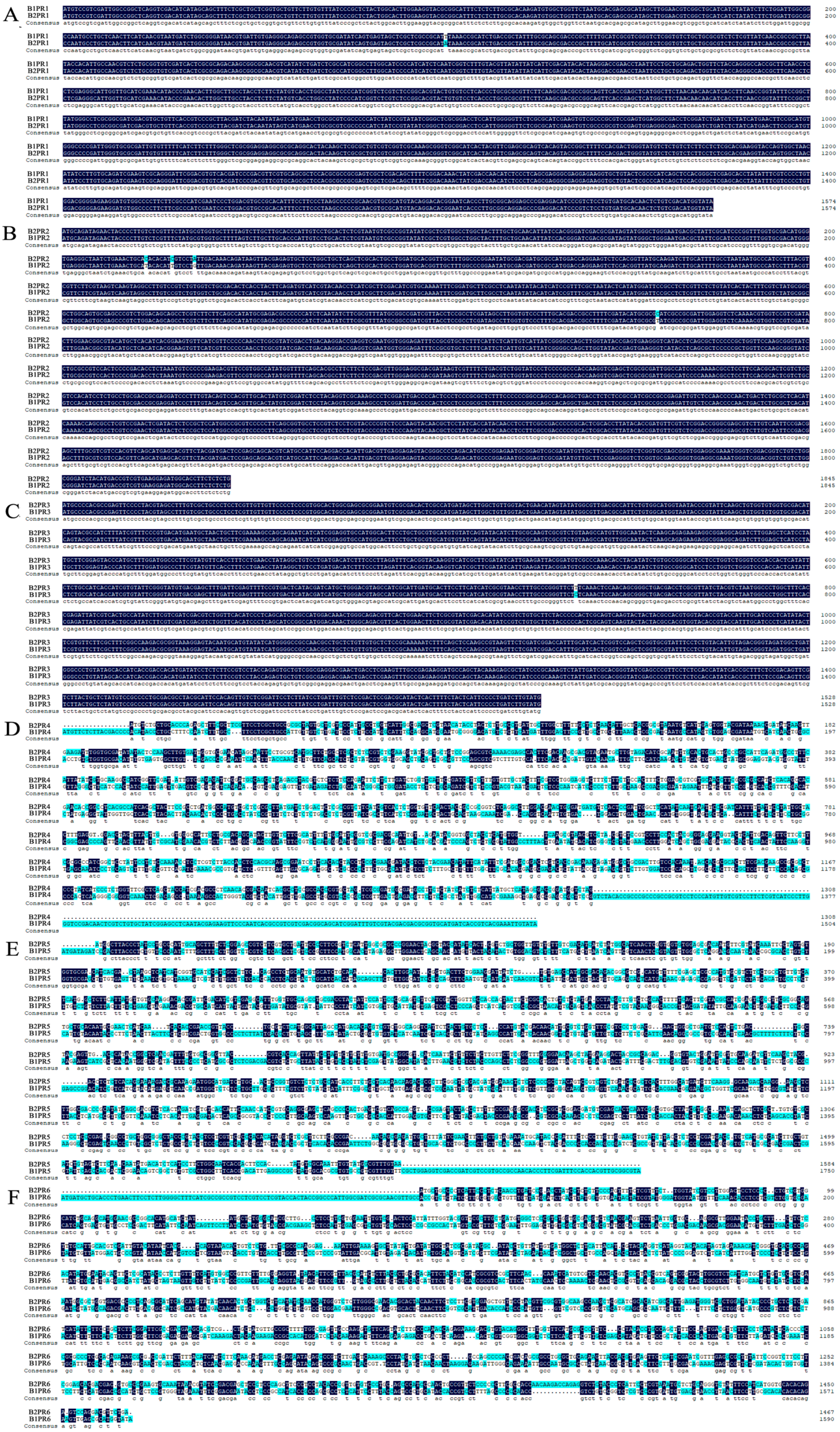
A
B1PR1
B2PR1
Consensus
B
B2PR2
B1PR2
C
B2PR3
B1PR3
D
B2PR4
B1PR4
E
B2PR5
B1PR5
F
B2PR6
B1PR6

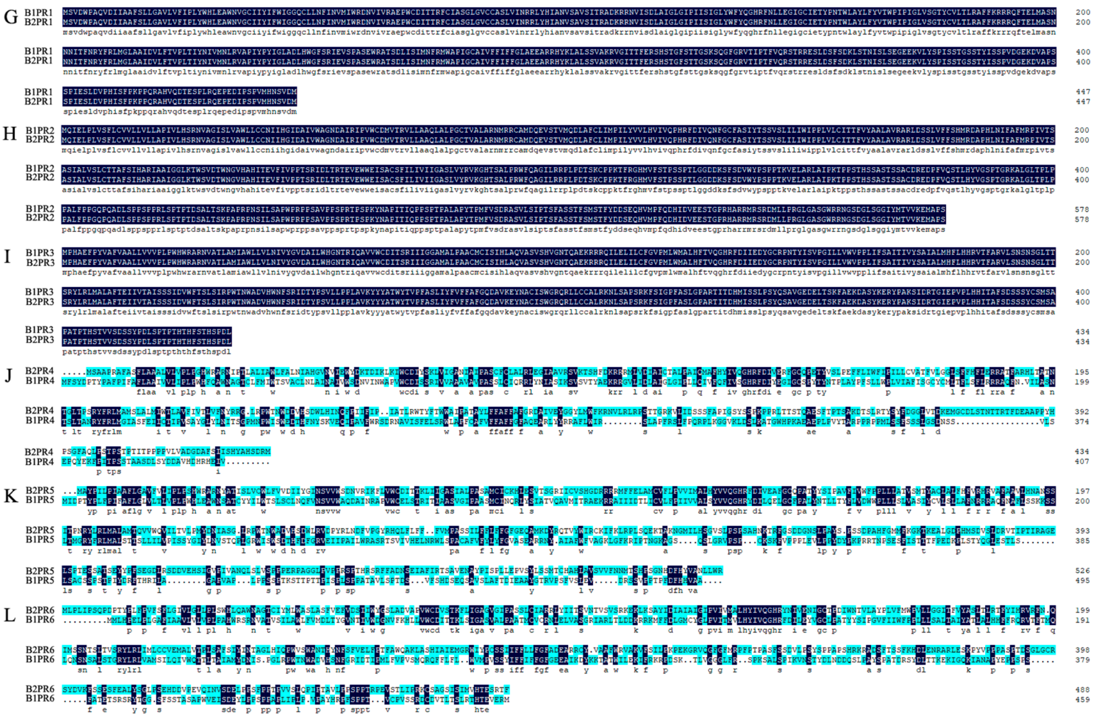

Figure S6: Alignment of *PR* genes of mating loci *B1* and *B2*. (**A**) DNA sequence alignment of *PR1* of mating loci *B1* and *B2*. (**B**) DNA sequence alignment of *PR2* of mating loci *B1* and *B2*. (**C**) DNA sequence alignment of *PR3* of mating loci *B1* and *B2*. (**D**) DNA sequence alignment of *PR4* of mating loci *B1* and *B2*. (**E**) DNA sequence alignment of *PR5* of mating loci *B1* and *B2*. (**F**) DNA sequence alignment of *PR6* of mating loci *B1* and *B2*. (**G**) Protein sequence alignment of *PR1* of mating loci *B1* and *B2*. (**H**) Protein sequence alignment of *PR2* of mating loci *B1* and *B2*. (**I**) Protein sequence alignment of *PR3* of mating loci *B1* and *B2*. (**J**) Protein sequence alignment of *PR4* of mating loci *B1* and *B2*. (**K**) Protein sequence alignment of *PR5* of mating loci *B1* and *B2*. (**L**) Protein sequence alignment of *PR6* of mating loci *B1* and *B2*.
